# Identification of the Core Set of Carbon-Associated Genes in a Bioenergy Grassland Soil

**DOI:** 10.1371/journal.pone.0166578

**Published:** 2016-11-17

**Authors:** Adina Howe, Fan Yang, Ryan J. Williams, Folker Meyer, Kirsten S. Hofmockel

**Affiliations:** 1 Department of Agricultural and Biosystems Engineering, Iowa State University, Ames, IA, 50011, United States of America; 2 Mathematics and Computer Science Division, Argonne National Laboratory, Argonne, Illinois, 60439, United States of America; 3 Department of Ecology and Evolutionary Biology, Iowa State University, Ames, IA, 50011, United States of America; 4 Pacific Northwest National Laboratory, Richland, WA, 99352, United States of America; Loyola University Chicago, UNITED STATES

## Abstract

Despite the central role of soil microbial communities in global carbon (C) cycling, little is known about soil microbial community structure and even less about their metabolic pathways. Efforts to characterize soil communities often focus on identifying *differences* in gene content across environmental gradients, but an alternative question is what genes are *similar* in soils. These genes may indicate critical species or potential functions that are required in all soils. Here we identified the “core” set of C cycling sequences widely present in multiple soil metagenomes from a fertilized prairie (FP). Of 226,887 sequences associated with known enzymes involved in the synthesis, metabolism, and transport of carbohydrates, 843 were identified to be consistently prevalent across four replicate soil metagenomes. This core metagenome was functionally and taxonomically diverse, representing five enzyme classes and 99 enzyme families within the CAZy database. Though it only comprised 0.4% of all CAZy-associated genes identified in FP metagenomes, the core was found to be comprised of functions similar to those within cumulative soils. The FP CAZy-associated core sequences were present in multiple publicly available soil metagenomes and most similar to soils sharing geographic proximity. In soil ecosystems, where high diversity remains a key challenge for metagenomic investigations, these core genes represent a subset of critical functions necessary for carbohydrate metabolism, which can be targeted to evaluate important C fluxes in these and other similar soils.

## Introduction

Soil microbial communities are of critical importance; they influence nutrient availability, decomposition rates, greenhouse gas emissions, soil fertility, and agricultural production [1–3]. Despite decades of research, we still know very little about soil microbial community structure and functioning, especially in agricultural soils. Sequencing-based approaches, particularly metagenomics, have greatly enhanced the resolution at which we can investigate genes contained within soil microbial communities [4–6]. Nonetheless we are becoming increasingly aware that the incredible diversity present in soils requires very deep sampling to capture (estimated Terabasepairs of sequencing) [7,8]. Efforts to characterize soil communities often focus on identifying *differences* in gene content across environmental gradients (e.g., genes present under varying land use history, nutrient loads, soil moisture content) [6,9–11]. An alternative question is what genes are *similar* in soils? In other words, is there a core set of sequences, genes, or functions that are present in *all* soils? Are the core genes found in one soil type, field, or even plot representative in soils? A similar effort in the gut microbiome environment identified gut-associated genes shared between multiple humans (124 European individuals) and provided a transformative reference gene set describing the minimal gut metagenome among these individuals and its encoded functions [12]. The microbial diversity in soils is magnitudes higher than the gut microbiome [13], suggesting that a soil core, if present, would be much smaller. We explore the presence of core gene sequences in a single experimental field and evaluate the insight it provides for soil function.

A single field site was selected for characterization of a soil core. Within a field, high levels of local spatial variation of microbial community structure have been observed [14–16]. Consequently, our study focused on the identification of a plot scale core soil microbial community in fertilized prairie (FP) whole soil (WS) metagenomes from a single experiment. The resulting soil core was also compared to several other soil metagenomes, including soil aggregate metagenomes from the same plot (e.g., originating from sieved partitions of the same FP WS), metagenomes from soils located nearby, and publicly available soil metagenomes. We expect that genes that are ubiquitously present in multiple soils may represent functions that are critical to soil processes. To evaluate the functions represented in our soil core, we identified genes associated with carbon (C) cycling and evaluated their contributions to microbial biomass synthesis and decomposition in these soils.

## Materials and Methods

Samples were collected with permission from the Committee for Agricultural Development, a nonprofit organization in Iowa that owns the property.

### Study site

Soil was collected from the Iowa State Comparison of Biofuel Systems (COBS) experimental site located on the South Reynoldson Farm in Boone County, IA (41°55'14.42"N, 93°44'58.96"W); see [17] for a detailed site description. Soils consisted of loams in the Nicollet (Fine-loamy, mixed, superactive, mesic Aquic Hapludoll) and Webster (Fine-loamy, mixed, superactive, mesic Typic Endoaquoll) series with less than 3% slope. Sand content ranged from 27% to 53% across the site and clay content ranged from 17% to 32%. In the 5 years prior to sampling, average growing season precipitation at the site was 91.8 cm and mean annual temperature was 9°C. Four replicate blocks contain four plots (27 x 61 m^2^) of each planting treatment in a randomized complete block design. The present study includes samples from plots planted with fertilized native tallgrass prairie (31 species). Soil cores (5.5 cm x 10 cm) were collected in July 2012 as described in [17]. Subsamples of soil were separated into soil aggregate fractions by an optimal sieving method prior to DNA sequencing. Biogeochemical analyses of these samples has been previously reported [18,19].

### DNA extraction and library preparation

For each soil sample, DNA was extracted from 0.25 g of soil by using MoBio PowerLyzer PowerSoil DNA Isolation Kit (MoBio, Carlsbad, CA). DNA was quantified using Nanodrop and approximately 1 μg of DNA per sample was used for metagenomic sequencing. Metagenome libraries were prepared with IntegenX PrepX DNA Library Kit with 180 bp overlapping inserts and subsequently size-selected prior to sequencing on an Illumina HiSeq2000. Library preparation and sequencing were performed at Argonne National Laboratory (Argonne, IL).

### Assembly and coverage of soil metagenome

An assembly of all soil metagenomes available from this site was used to generate a reference set of contigs for this study as previously described in [7]. All sequencing reads originating from FP samples (both WS and varying sizes of soil aggregates, n = 20) were combined and assembled (sequencing reads available for all data in MG-RAST, see S1 and S2 Tables) to create a cumulative reference metagenome. This reference was used to identify shared core contig sequences among all FP WS metagenomes. Prior to assembly, Illumina adapters were trimmed with Trimmomatic (v0.27, [20]) using Illumina TruSeq2-PE with threshold of seed mismatches, palindrome clip threshold, and simple clip threshold as 2, 30, and 10, respectively. Remaining paired end sequences were merged with PANDAseq [21]. The resulting sequences were normalized using the *khmer* package [22–24] and methods previously described in [7] with the following parameters: -k 20 -C 10 -N 4 -x 100e9. High abundance k-mers with coverage > 50 were trimmed from sequences, and the remaining sequences were partitioned as previously described in [7,25] with the following parameters: -k 32 -N 4 -x 80e9. Assembly was performed with the Velvet assembler (v 1.2.10, [26]) using odd k-mer lengths from 33 to 65. Resulting assembled contiguous sequences (contigs) were merged as described previously [7] using CD-HIT (v4.6, [27,28] and minimus2 (Amos v3.1.0, [29])

Abundances of contigs associated with each sample was estimated through the alignment of sequencing reads with assembled contigs using Bowtie2 (v2.0.5, default parameters, [30]). Coverage of each contig was estimated as the maximum base pair coverage of the assembled sequence, requiring a minimum of coverage length of 100 bp. This abundance estimation was intentionally chosen to be liberal, intending to capture representative core contigs even at low abundance in individual samples. To be identified as present in *all* samples, contigs were required to be present at 5 or greater base pair coverage in all four local soil metagenomes. For core contigs, the MG-RAST automated annotation system was used to identify associated function of assembled contigs (MG-RAST ID 4519723.3) [31]. In order to standardize samples with various sampling depths, the total number of single-copy *recA* genes in each sample was estimated, using annotations from MG-RAST, requiring a sequence alignment (E-value < 1e-5) to *recA* (Subsystem ID SS04542). The coverage of each gene was estimated from the base pair estimated coverage of its originating assembled contig divided by the total estimated *recA* genes identified in each sample. To identify carbon-associated genes, all assembled contigs were compared to known proteins in the Carbohydrate Active Enzyme (CAZy) database [32] using NCBI BLAST (v2.2.25, blastx), requiring an E-value ≤ 1e-5. The best scoring alignment to the CAZy database for each sequence was used for characterization (both function and taxonomic origin). If multiple annotations shared identical best score alignments, one was chosen at random. The resulting set of CAZy-associated assembled contigs comprised the dataset referred to as the cumulative FP-CAZy metagenome. Annotations associated with each contig as well as analysis performed in this study can be found at https://github.com/germs-lab/carbon-core-soil-paper.

### Characterization of the fertilized prairie whole soil carbon core community

To identify the genes present in all of the available metagenomes of the FP WS, we defined the *core* carbohydrate-associated contigs as sequences that were present a minimum abundance of 5 or greater base pair coverage in each of the four WS field replicate metagenomes; these core contigs are hereafter referred to as the FP-CAZy core. As we focused on carbohydrate-associated genes, only contigs that shared sequence similarity to a known protein within the CAZy database were considered.

To examine the content of the FP-CAZy core, a phylogenetic tree (based on bacterial and archaeal 16S rRNA genes) was constructed for bacteria associated with FP-CAZy core contigs (S1 Fig). As core proteins cannot be aligned easily to construct a phylogenetic tree, representative 16S rRNA genes associated with phyla associated with CAZy genes were obtained. Core sequences were searched against GenBank (release 198.0) from which organisms with full genomes were identified. Bacterial and archaeal 16S rRNA genes were extracted from these full genomes to build the phylogenetic tree. For microorganisms containing multiple 16S rRNA genes, the first gene sequence identified in the GenBank record was selected as representative. The 16S rRNA gene sequences were aligned by using RDP (Ribosomal Database Project, release 11, [33]) Aligner. MEGA 5.2 (Molecular Evolutionary Genetics Analysis, [34]) was used to construct the phylogenetic tree. Specifically, the phylogeny was inferred using maximum likelihood heuristic method (nearest neighbor interchange) with a general time reversible nucleotide substitution model (discrete gamma distribution with 5 categories and allowing the presence of invariant sites). The phylogeny was tested using the boostrap method (999 times). For every microorganism in the phylogenetic tree, the abundance of associated FP-CAZy core contigs associated to that phyla (standardized against the recA gene abundance) was also calculated. For each metagenome CAZy annotations were grouped into six CAZy classes, glycosyltransferases (GT), glycoside hydrolases (GH), carbohydrate esterases (CE), carbohydrate-binding modules (CB), polysaccharide lyases (PL) and unknown. The abundance of each CAZy class was summed based on phylogenetic identification within the same sample, and averaged across 4 replicates (at 95% confidence intervals).

The resulting FP-CAZy core contigs were compared to other soil metagenomes. Within the COBS experimental site, these other metagenomes included soil aggregate fractions isolated from FP WS (n = 14), as well as microaggregates isolated from unfertilized prairie (n = 4) and continuous corn (n = 2) cropping systems (S2 Table). FP-CAZy core sequences were considered present in these samples if identified in at least one field replicate with 5 or greater base pair coverage. To assess the relevance core sequences more broadly, we analyzed several public soil metagenomes (Fierer et al 2012, Howe et al 2014), where presence of FP-CAZy core sequences were considered to be present if sequence similarity of the best scoring BLAST alignments of core sequence to metagenome reads had a minimum E-value score of 1e-5, length of 70, and identity of 70%.

## Results

### Characterization of the FP-CAZy core

The total size of each FP WS replicate metagenome ranged from 4.3 to 20.5 Gbp (average 10.3 ± 7.1 Gbp). Within these metagenomes, a total of 226,887 contigs were identified with shared sequence similarity to known CAZy proteins. Among these contigs, a total of 11,193 contigs were identified as present in all four replicates (at varying abundances). Requiring a minimal abundance of greater than 5-fold bp coverage, a total of 911 sequences were present in all four metagenomes and comprised the FP-CAZy core metagenome (representing 499,329 bp of 41.4 Gbp). Given the conservative requirements for core sequences, these represent a lower bound estimate of shared sequences within the field replicated FP metagenomes. To evaluate the effects of sequencing depth, we estimated the total number of core k-mers in subsets of the four WS metagenomes, 100,000 to 100 million reads (278 to 539 million unique k-mers average, n = 4 bootstraps). We found that the total core k-mers comprised 4.8 to 8.9% of total unique k-mers, suggesting that with more sequencing, the core size continue to grow linearly. The sequencing depth represented within this study (minimum 4.3 Gbp) is greater than the average sequencing depth of 1.67 Gbp (median 0.47 Gbp) of 1,093 public soil-associated metagenomes in MG-RAST (October 27, 2015, material = peat soil, sediment, soil, agricultural soil, alpine soil, arable soil, bulk soil, clay soil, farm soil, grassland soil, lawn soil, leafy wood soil, paddy field soil, rhizosphere, sandy sediment, volcanic soil, and xylene contaminated soil). Even with this above average sequencing effort, we were able to identify only 911 core sequences among the four FP metagenomes. When we further limited our analysis to CAZy-associated proteins related to bacteria, archaea, viruses, and fungi, our core consisted of a total of 843 contigs shared between all four FP replicates.

**Fig 1:**
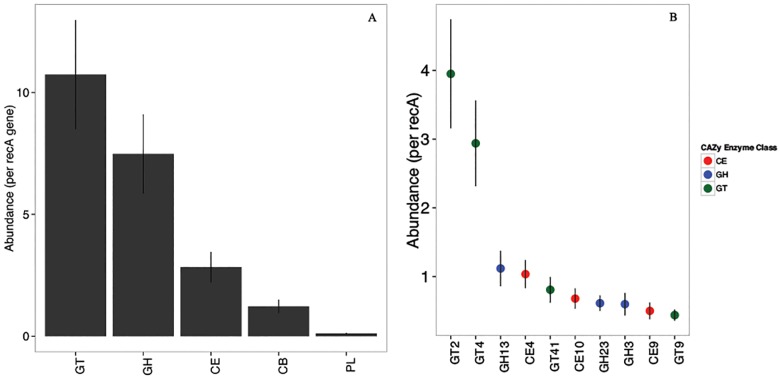
Functional profile (mean ± SE) of CAZy enzyme classes (A) and 10 most abundant enzyme families (B) represented in FP-CAZy core sequences (GT, glycosyltransferase; GH, glycoside hydrolase; CE, carbohydrate esterase; CB, carbohydrate-binding module; PL, polysaccharide lyase).

**Fig 2:**
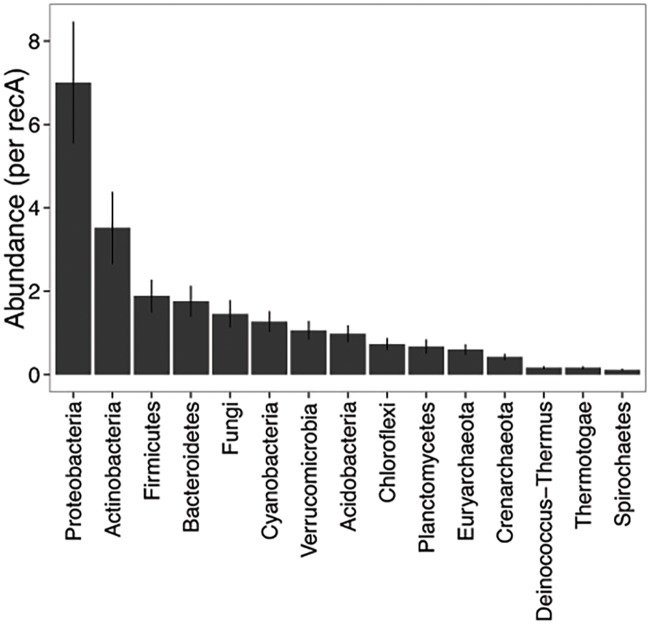
Abundance of phyla represented in the FP CAZy core (mean ± SE; n = 4).

We next explored the content of our core, evaluating the putative functions and taxonomy of proteins sharing similarity to core contigs. Functions and taxonomy associated with core sequences were quantified in their prevalence (number of unique occurrences within the core) and relative abundance (cumulative abundance). Overall, the FP-CAZy core of 843 contigs represented five major enzyme classes and 99 enzyme families. The average relative abundance of proteins indicated GT > GH > CE > CB > PL (Fig 1A, S1 Fig). Among these, the most abundant enzyme families included GT2, GT4, and GH13, which were associated with a total prevalence of 145, 109, and 38 core sequences and present at estimated average abundances of 4.0, 2.9, and 1.1 copies per *recA* sequence, respectively (Fig 1B). The most represented taxa of the FP-CAZy core were similar to Proteobacteria, Actinobacteria, and Firmicutes, related to a total prevalence count of 266, 120, and 70 unique assembled sequences, respectively. The relative abundances of phyla associated with core sequences revealed diverse membership (Fig 2), with sequences associated with Proteobacteria broadly represented in association with enzymes GT, GH, and CE (S1 Fig). Fungal sequences associated with GH and CE enzymes were also abundant. A few core enzyme classes were represented by only a single phylum, the most abundant classes including GH94 (37 copies/1000 *recA*, Proteobacteria) and GH76 (29 copies/100 *recA*, Fungi).

Because the identification of core sequences required similarity to protein encoding sequences within the CAZy database, we evaluated potential biasing of the core from the CAZy database itself. We randomly selected 50,000 proteins each from the CAZy database and the cumulative metagenome to generate a random set of CAZy classes and associated taxonomy. The simulated random distribution was performed 1000 times to compare to the observed CAZy classes and phylogeny of the core sequences. We found that protein distributions were significantly different for both CAZy class and phyla (ANOVA, p < 0.05), suggesting that observations here not the result of database bias.

### Representation of FP-CAZy core in cumulative FP and other soil metagenomes

The total number of FP-CAZy core sequences represent only 0.4% of all CAZy proteins identified in the cumulative FP metagenomes (843 out of 226,887 unique sequences). With greater sequencing depth, we would expect this number to increase significantly. By abundance, the core comprises ~1% of the most abundant (≥ 5-fold bp coverage) CAZy-associated sequences. We compared core functions and taxonomy to those all observed functions and taxa in the FP metagenomes. The distributions of the abundances of identified CAZy enzyme classes in the core and cumulative FP metagenome were similar though the relative abundances of CB and GT differed by up to 4% (S3 Fig). We observed that at the enzyme family level, abundances in the core and cumulative datasets contrasted, with the exception of GT2 and CE10 (p-value > 0.2). Similarly, the distribution of phyla-associated with dominant enzyme families (e.g., GT2, GH13, CE10, and GT4) was significantly different between the core and cumulative metagenomes (S4 Fig). For example, proteins associated with GT2 originating from Actinobacteria (14 sequences) and Firmicutes (6 sequences) were significantly enriched in the core.

**Fig 3:**
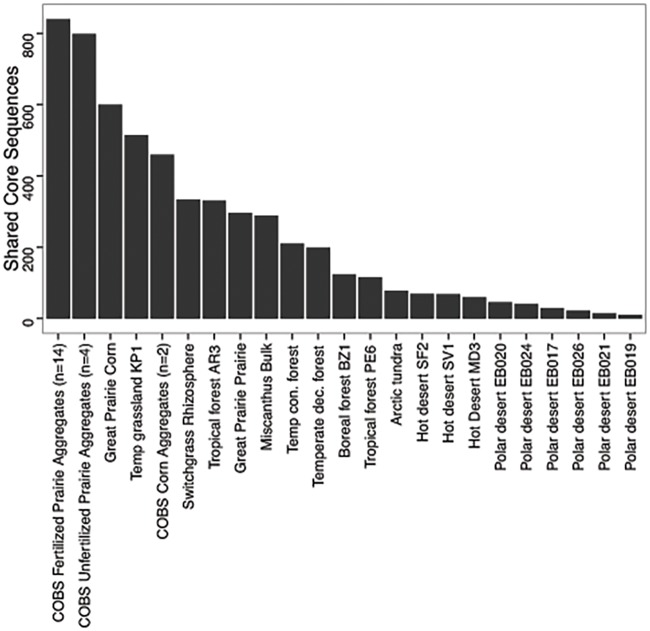
Number of shared fertilized prairie metagenome core sequences in global soil metagenomes sharing sequence similarity (E-value 1e-5).

**Fig 4:**
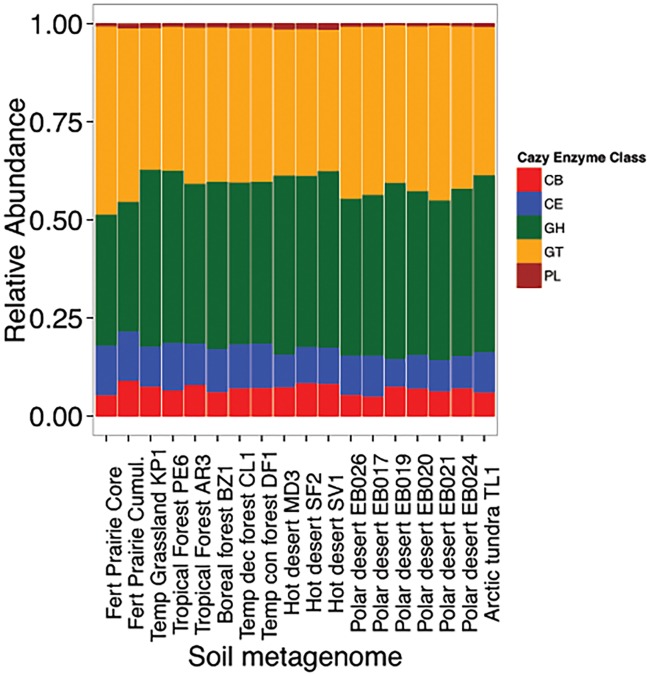
Functional distribution of the presence of CAZy enzyme classes in global soil metagenomes.

Comparison of the FP-CAZy core to metagenomes of FP aggregates as well as adjacent corn and unfertilized prairie aggregates (Bach and Hofmockel 2014) revealed substantial sequence similarity. The large majority of FP-CAZy core sequences, 840 out of 843, were also identified within FP soil aggregate fractions (sieved fractions of whole soil samples). In adjacent soil samples (microaggregates of unfertilized prairie (n = 4) and corn (n = 2) fields, a total of 792 and 600 FP-CAZy core sequences were observed in unfertilized prairie and corn metagenomes, respectively. Comparing the FP-CAZy core to other globally distributed soil metagenomes (Fig 3, S2 Table) revealed the most shared sequences (e.g., sequence similarity) with other grassland (405 ± 109) and agricultural soils (535 ± 95) and fewer shared core sequences with forest (196 ± 39), tundra (78), and desert (40 ± 8) soils. However, at the functional level (as opposed to sequence similarity), the relative distribution of CAZy enzyme classes was broadly similar (Fig 4).

## Discussion

The soil represents arguably the most challenging environment to access with modern molecular microbial ecology. Its high diversity and spatial heterogeneity, even at the meter scale, make it difficult to sample and characterize. Despite these difficulties, the importance of microbial communities in terrestrial biogeochemistry and ecosystem C- cycling are well agreed upon [35,36]. Understanding the functional capacity of soil microorganisms remains an important goal for understanding ecosystem health and stability [37]. We explored the insights a minimal local soil core metagenome could provide for identifying key enzymes, microorganisms, and ecological functions related to soil C cycling. Among all C-cycling enzymes identified in our soil metagenomes, the CAZy-associated soil core represented only 0.4% of genes. Despite representing only a fraction of all genes observed in FP metagenomes, the identified FP-CAZy core shared similar functions to whole metagenomes, supporting our hypothesis that core sequences represent a set of minimum C-cycling functions necessary in FP soils. The most dominant functions identified within the core and cumulative metagenomes were GT2 and GT4, which are involved in the formation of cell wall polysaccharides of diverse organisms including bacteria, archaea, fungi, and plants, as well as numerous biological processes such as pathogen protection, intercellular signaling, and biofilm production [38]. In the core, these enzymes have been observed as originating from Proteobacteria. Sequences similar to Bacteroidetes and Actinobacteria dominated the GT2 family in core functions, while Verrucomicrobia and Euryarchaeota genes were prevalent in GT4 family. We observe that broad membership may provide core enzymes in soils, supporting previous observations that high biodiversity may help to stabilize carbon cycling [39,40].

We found that genes associated with amylolytic enzymes (GH13) were highly prevalent in the core, highlighting the central importance of breakdown and utilization of starch and related oligo- and polysaccharides. In general, genes known to be associated with GH13 contribute to trehalose synthesis, a compound that is used by both plants and fungi to store carbon and energy, as well as protecting bacterial cells from physical and chemical stresses [41]. Abundant core sequences associated with GH13 are similar to genes of Proteobacteria, Actinobacteria, and Planctomycetes, suggesting that these organisms may play a central role in starch utilization. Although Proteobacteria and Actinobacteria are commonly associated with C cycling in soil, Planctomycetes have only recently been identified in agricultural soils [42], tundra soils [43], and Arctic peats [44], demonstrating its global presence, and potential functional importance in soils. Additionally, Planctomycetes have been associated with decomposition of cellulose within agricultural soils [45], making this a noteworthy phylum for further investigations focused on soil C cycling.

Genes that were enriched in the core relative to the cumulative metagenome were hypothesized to play critical roles in soil C-cycling. These genes included sequences sharing similarity to GH13 associated Actinobacteria and Planctomycetes, GT4 associated Actinobacteria, Firmicutes, Euryarchaeota, and Verrucomicrobia, GT2 associated Actinobacteria and Firmicutes, and CE10 associated Fungi and Proteobacteria sequences. These enriched core functions represented a relatively small diversity of all observed carbon related functions. Enriched core genes comprised 3% of cumulative GT2-associated Actinobacteria and Firmicutes proteins; 3% of GT4-associated Actinobacteria, Firmicutes, Euryarchaeota, and Verrucomicrobia proteins; 14% of CE10-associated Proteobacteria and Fungi proteins; and 5% of GH13-associated Actinobacteria and Planctomycete proteins. Their prevalence in multiple soils suggest that amongst diverse functions and memberships, these genes and bacteria are critical for C-cycling.

Another interesting observation within the core was the presence of ubiquitous and abundant sequences associated with CE, in particular CE10. In general, CE genes act on plant polysaccharides to degrade acetylated plant hemicelluloses [46] and are commonly clustered with GHs in operons or regulons and are co-expressed to decompose esterified polysaccharides of plant cell walls. Little is known about CE10, which is associated with colinesterase type enzyme. Soil substrates associated with CE, α- and β-glycerophosphates and choline-P, have been identified as degradation products of phospholipids of cellular membranes during NMR analysis [47–49] and have been shown to cycle and accumulate in agricultural soils [50]. The observed CE10 presence in the core supports the premise that cell membranes may be an important soil substrate, and CE hydrolases may be a potential target for quantifying the importance of microbial cell wall turnover, which is a pressing question given the putative importance of microbial necromass to soil C storage [51,52].

At a broad level, the functions encoded by the core are observed in global soils, ranging from those originating from agriculture to deserts. However, at the strain level (e.g., sequence variation), our core was significantly more represented in soils from similar land-use and geography. Within the same large-scale field experiment, we found that independent of crop selection or management practices (corn and unfertilized prairie), multiple soil metagenomes shared a large majority of core sequences (97%). In soils originating from Iowa but located about 60 miles SE from the FP site, core sequences were less prevalent compared to local samples (33% in prairie and 68% in corn). In contrasting soils, such as deserts and forests, significantly fewer FP-CAZy core sequences could be identified, suggesting that the soil environment, which can be geographically specific, is important for defining a soil core and its functional potential. This result has been reported previously where soil type was the critical driver of microbial community compositional differences [53,54]. For metagenomic studies, this observation has implications for the genomic or functional level that should be compared between studies. We observe that genes encoding for functions (e.g., enzyme classes) are largely similar in global soils but find that core sequences are not broadly representative, at least at the current sequencing depths being used to study oil microbiomes.

Sequencing-based approaches for studying the soil microbial communities continue to increase in volume and in complexity (e.g., metaproteomics and metabolomics). Our results present a challenge that is confronting the study of soil ecosystems. At a local scale (soils originating from the same experimental plot), greater than average sequencing depth for current soil studies, and focusing on genes encoding for carbon cycling functions, we are able to identify less than a 1% signal of sequences being shared among multiple metagenomes. In contrast, the gut microbiome identified that 40% of genes was shared within at least half of the 124 individuals studied [12]. These results reaffirm that we are still only beginning to sample the immense diversity in these soils and are far from identifying a minimal soil core microbiome, at least at the gene level. Our results also emphasize the need to consider varying scales of characterization when comparing soil microbial communities. Given the unique nature of soil, we have a strong need to evaluate new methods for binning soil sequences include protein clustering (e.g., operational protein units analogous to operational taxonomic units from sequenced 16S rRNA amplicons), co-occurrence networks and interactions, and improved hierarchy in functional annotations. Continued efforts to us our identified targets to expand what is known about the minimal genes encoding functions in soil will be helpful to identify critical community processes within complex metagenomes and can serve as the framework with which to deconstruct and understand the high diversity of microbial soil communities.

## Supporting Information

S1 Fig Phylogenetic tree of taxonomic origins of CAZy proteins most similar to core genes (using available 16S rRNA genes). The diamonds on the maximum likelihood phylogenetic tree indicate branches with bootstrap values greater than 80. CAZY gene abundance was calculated across four whole-soil replicates and the error bars represent 95% confidence intervals.
(EPS)

S2 Fig Abundance of phyla represented in enzyme families GH, GT, CB, and CE associated with the fertilized prairie core metagenome. (EPS)

S3 Fig Relative abundance of CAZy enzyme families and classes in fertilized prairie metagenome core and the cumulative fertilized prairie metagenome. (EPS)

S4 Fig Abundance of phyla associated with enzymes in fertilized prairie metagenome core and the cumulative fertilized prairie metagenome. (EPS)

S1 Table Sequencing summary of fertilized prairie whole soil metagenomes. (DOCX)

S2 Table Number of shared core sequences among various soil metagenomes. Number of replicates is one unless otherwise indicated.
(DOCX)
