# Supplementary material for: Identification of the Core Set of Carbon-Associated Genes in a Bioenergy Grassland Soil
Source: PLoS One. 2016 Nov 17;11(11):e0166578. doi: 10.1371/journal.pone.0166578 (PMC5113961; doi:10.1371/journal.pone.0166578)
Supplement: S1 Table — (DOCX) [file pone.0166578.s005.docx]

S1 Table. Sequencing summary of fertilized prairie whole soil metagenomes.

| Sample ID | MG-RAST ID | Total Sequencing (bp) | Total Number of Reads |
| --- | --- | --- | --- |
| PF15-WS-July2012 | 4509403.3 | 20,524,028,604 | 203,208,204 |
| PF32-WS-July2012 | 4511177.3 | 7,747,986,740 | 76,712,740 |
| PF41-WS-July2012 | 4511166.3 | 4,315,928,364 | 42,731,964 |
| PF23-WS-July2012 | 4509407.3 | 8,778,023,120 | 86,911,120 |
