# Supplementary material for: Identification of the Core Set of Carbon-Associated Genes in a Bioenergy Grassland Soil
Source: PLoS One. 2016 Nov 17;11(11):e0166578. doi: 10.1371/journal.pone.0166578 (PMC5113961; doi:10.1371/journal.pone.0166578)
Supplement: S2 Table — Number of replicates is one unless otherwise indicated. (DOCX) [file pone.0166578.s006.docx]

S2 Table. Number of shared core sequences among various soil metagenomes. Number of replicates is one unless otherwise indicated.

| Samples | Soil Ecosystem | MG-RAST IDs | Number of Shared Core Sequences |
| --- | --- | --- | --- |
| COBS Fertilized Prairie Aggregates (n=14) | Agricultural | 4509396.3-4509402.3; 4509405.3-4509406.3; 4511167.3- 4511168.3, 4511170.3-4511172.3 | 840 |
| COBS Unfertilized Prairie Aggregates (n=4) | Agricultural | 4514436.3; 4514437.3; 4514438.3; 4514439.3 | 798 |
| Great Prairie Corn | Agricultural | 4504797.3 | 600 |
| Temp grassland KP1 | Grassland | 4477804.3 | 514 |
| COBS Corn Aggregates (n=2) | Agricultural | 4511173.3; 4511176.3 | 460 |
| Switchgrass Rhizosphere | Agricultural | 4465936.3 | 334 |
| Tropical forest AR3 | Forest | 4477875.3 | 331 |
| Great Prairie Prairie | Grassland | 4504798.3 | 296 |
| Miscanthus Bulk | Agricultural | 4465947.3 | 289 |
| Temp con. forest | Forest | 4477899.3 | 211 |
| Temperate dec. forest | Forest | 4477877.3 | 199 |
| Boreal forest BZ1 | Forest | 4477876.3 | 124 |
| Tropical forest PE6 | Forest | 4477807.3 | 116 |
| Arctic tundra | Tundra | 4477874.3 | 78 |
| Hot desert SF2 | Desert | 4477872.3 | 70 |
| Hot desert SV1 | Desert | 4477873.3 | 69 |
| Hot Desert MD3 | Desert | 4477805.3 | 60 |
| Polar desert EB020 | Desert | 4477902.3 | 46 |
| Polar desert EB024 | Desert | 4477904.3 | 41 |
| Polar desert EB017 | Desert | 4477900.3 | 29 |
| Polar desert EB026 | Desert | 4477803.3 | 23 |
| Polar desert EB021 | Desert | 4477903.3 | 15 |
| Polar desert EB019 | Desert | 4477901.3 | 10 |
